# PMCA4 (ATP2B4) mutation in familial spastic paraplegia causes delay in intracellular calcium extrusion

**DOI:** 10.1002/brb3.321

**Published:** 2015-02-16

**Authors:** Philip Wing-Lok Ho, Shirley Yin-Yu Pang, Miaoxin Li, Zero Ho-Man Tse, Michelle Hiu-Wai Kung, Pak-Chung Sham, Shu-Leong Ho

**Affiliations:** 1 Division of Neurology, Department of Medicine, University of Hong KongHong Kong, China; 2 Research Centre of Heart, Brain, Hormone and Healthy Aging, University of Hong KongHong Kong, China; 3 Department of Psychiatry, University of Hong KongHong Kong, China; 4 Centre for Reproduction, Development and Growth, University of Hong KongHong Kong, China; 5 Centre for Genomic Sciences, University of Hong KongHong Kong, China

**Keywords:** ATP2B4, calcium transient, Familial spastic paraplegia, neurodegeneration, PMCA

## Abstract

**Background:**

Familial spastic paraplegia (FSP) is a heterogeneous group of disorders characterized primarily by progressive lower limb spasticity and weakness. More than 50 disease loci have been described with different modes of inheritance. Recently, we described a novel missense mutation (c.803G>A, p.R268Q) in the plasma membrane calcium ATPase (PMCA4, or ATP2B4) gene in a Chinese family with autosomal dominant FSP. Further to this finding, here we describe the functional effect of this mutation.

**Methods:**

As PMCA4 removes cytosolic calcium, we measured transient changes and the time-dependent decay of cytosolic calcium level as visualized by using *fura-2* fluorescent dye with confocal microscopy in human SH-SY5Y neuroblastoma cells overexpressing either wild-type or R268Q mutant PMCA4.

**Results:**

Overexpressing both wild-type and R268Q PMCA4 significantly reduced maximum calcium surge after KCl-induced depolarization as compared with vector control cells. However, cells overexpressing mutant PMCA4 protein demonstrated significantly higher level of calcium surge when compared with wild-type. Furthermore, the steady-state cytosolic calcium concentration in these mutant cells remained markedly higher than the wild-type after SERCA inhibition by thapsigargin.

**Conclusion:**

Our result showed that p.R268Q mutation in PMCA4 resulted in functional changes in calcium homeostasis in human neuronal cells. This suggests that calcium dysregulation may be associated with the pathogenesis of FSP.

## Introduction

Familial spastic paraplegia (FSP) is a clinically and genetically heterogeneous group of diseases characterized by progressive lower limb spasticity and weakness, and is classified according to phenotype, mode of inheritance, and the mutated gene (Blackstone 2012). Pure FSP is characterized by progressive lower limb weakness and spasticity and may be associated with urinary urgency, mild impairment of vibration sense, and proprioception. The upper limbs are spared and there is no bulbar dysfunction. Complex FSP is characterized by additional manifestations such as cognitive impairment, epilepsy, cerebellar ataxia, extrapyramidal disturbances, optic atrophy, and peripheral neuropathy. Neuroimaging may show white matter lesions, thin corpus callosum, and spinal cord atrophy. FSP can be inherited in an autosomal dominant, autosomal recessive, or X-linked fashion (Finsterer et al. 2012). Different mutations in the same gene can cause either pure or complex FSP, and intrafamilial phenotypic variability is high, greatly complicating the genetic diagnosis of FSP. Seventy-one forms of FSP (SPG1 to SPG48) have been described involving many gene loci (Novarino et al. 2014), with 20 or more loci associated with autosomal dominant FSP (Finsterer et al. 2012). The associated genes have been reported to be involved in organelle and microtubule dynamics, endoplasmic reticulum homoeostasis, transport, and signal transduction.

Recently, we described a novel causative mutation, c.803G>A, p.R268Q in the PMCA4 gene in a Chinese family with autosomal dominant FSP (Li et al. 2014). This mutation was predicted to be pathogenic based on multiple deleteriousness prediction algorithms. PMCA4 is known to have protein–protein interaction and shares similar cellular pathways with some known causal genes of FSP and spinocerebellar ataxias. Computational modeling of the mutant PMCA4 protein showed that the mutation, which is located in a protein aggregation-prone segment, may result in a higher folding free energy. In this study, we aimed to further study the functional impact of this mutation on calcium transient using human neuroblastoma cells. We demonstrated that the R268Q mutation significantly decreased the efficiency of suppressing depolarization-induced calcium overload in these cells when compared with cells overexpressing wild-type PMCA4.

## Materials and Methods

### Cloning of human PMCA4 transcript

Full-length human PMCA4 isoform b (Protein ID: ENSP00000350310) cDNA was amplified from total RNA extracted from HEK-293 cells. The gene-specific primers used were as followed: PMCA4b-forward, 5′-ATCGGATCCATGACGAACCCATCAGACC, and PMCA4b-reverse, 5′-ATCGCGGCCGCTCAAACTGATGTCTCTAGGCTCT. A mutated mega-primer, prepared using PMCA4b-forward and PMCA4b-M-reverse, 5′-CATCTGGCCAGAACCTT, along with PMCA4b-reverse was used to amplify the mutant form of PMCA4b (PMCA4b-mut, 803G>A) using full-length wild-type PMCA4b amplicon as template. The PCR products were then subcloned into mammalian expression vector, pcDNA3.1(+), and transformed into *E. coli* (DH5*α*). Positive clones containing either PMCA4b or mut-PMCA4b were confirmed with sequencing.

### Cell culture

Human neuroblastoma SH-SY5Y cells (CRL-2266; ATCC) were cultured in DMEM-F12 supplemented with 10% FBS, 2 mmol/L glutamine and 100 *μ*g/mL penicillin-streptomycin (Invitrogen, Hong Kong) at 37°C in humidified 5% CO_2_ atmosphere. Expression plasmids containing transcript of either wild-type or mutant PMCA4 were transfected into SH-SY5Y cells using Lipofectamine2000 (Invitrogen). Twenty-four hours after transfection, cells were selected using geneticin (G-418) at 250 *μ*g/mL until resistant clones were obtained.

### Real-time PCR

Total ATP2B4 mRNA expression was determined by quantitative RT-PCR. Total RNA was isolated and digested with DNase I as previously described (Ho et al. 2010). The OD_260/280_ ratio of extracted RNA was kept above 1.95 to ensure purity and integrity. Reverse transcription was performed using total RNA by AccuScript High Fidelity 1st Strand cDNA synthesis kit (Agilent Technology, Santa Clara, CA). Amplification reactions were performed using Power SYBR Green PCR Master Mix (Applied Biosystems) according to manufacturer's protocol. The primers for real-time PCR: PMCA4b-forward: GCAATACCTACCCGATCCCTG; PMCA4b-reverse: ACCGCATTGTTGTTTGTATTGG. GAPDH expression was used to normalize for mRNA loading. Each RNA sample was run in triplicate and repeated in at least three independent treatments.

### Western blot

Overexpression of PMCA4 was confirmed using SDS-PAGE western blotting. Equal amount of total cell lysates extracted in standard RIPA were subjected to polyacrylamide gel electrophoresis, followed by standard western blotting procedures. Antibodies against total PMCA4 (Pierce, MA1-914) and *β*-actin (Santa Cruz, sc-1615) were used to detect the proteins, respectively. Relative expression level of target proteins was determined by the band intensity as measured using densitometry.

### Intracellular calcium level [Ca^2+^]_i_ measurement

Human SH-SY5Y cells (2 × 10^5^ cells/well) were plated in 24-well plates coated with 100 or 10 *μ*g/ml poly-L-lysine (Sigma, St. Louis, MO) with DMEM/F12 at 37°C overnight for adherence. To determine the effects of PMCA4 mutation on the intracellular calcium transient, cells stably overexpressing either wild-type or mutant PMCA4 were labeled with 5 *μ*mol/L *fura2*-AM fluorescent dye (Invitrogen) in serum-free DMEM for 20 min in darkness at room temperature. After subsequent washes with Gey's balanced salt solution (GBSS), cells were incubated in GBSS for a further 10 min at 37°C to allow de-etherification of AM esters. Labeled cells were put on the stage of an Olympus inverted epifluorescence microscope (Olympus, Tokyo) and visualized using a 20 × objective. Images were collected by a CCD camera every 2 sec and analyzed using Olympus Cell R Imaging System (Olympus, Germany) at room temperature with excitation of 340 and 380 nm, and emission was measured at 510 nm. Ratio (R) of *F*_340_/*F*_380_ was obtained after subtracting background noises. Cells were allowed to stabilize for 20 sec and baseline ratios (*R*_o_) were obtained prior to addition of potassium chloride (KCl, 100 mmol/L) to induce membrane depolarization, or incubation with the SERCA inhibitor, thapsigargin (TG, 0.5 *μ*mol/L). The measurement continued for 4 min after addition of KCl or TG. The relative changes in intracellular calcium levels [Ca^2+^]_i_, indicated as normalized ratio (R/R_o_), were plotted against time. Intracellular concentration of calcium was calculated using the general formula, [Ca^2+^]i = Kd * Q(R-R_min_)/(R_max_-R). (*Kd* =183 nmol/L). *K*d is the dissociation constant for Ca^2+^ binding to the indicator, R is the fluorescence intensity ratio between emission at 340 and 380 nm with fura-2. Q is the ratio of minimum (*F*_min_) to maximum (*F*_max_) intensity at OD_380_. *F*_max_ was determined by exposing cells to 1.5 mmol/L Ca^2+^ and 5 *μ*mol/L ionomycin, and *F*_min_ was determined by the addition of 4 mmol/L EGTA and 5 *μ*mol/L ionomycin to cells.

### Statistical analysis

Statistical analyses were carried out using the Prism (GraphPad Software Inc., San diego, CA). Data are expressed as mean ± standard error mean (SEM) from at least three independent experiments. Statistical significance among individual groups was calculated by the Student's *t*-test. The time dependent decay in cytosolic calcium was assessed by analysis of extra sum-of-squares *F* test after fitting of one phase decay (*t*_1/2_). Differences were considered significant at a level of *P* < 0.05.

## Results

### R268Q mutant PMCA4 delayed calcium extrusion after induced calcium transient

**Figure 1:**
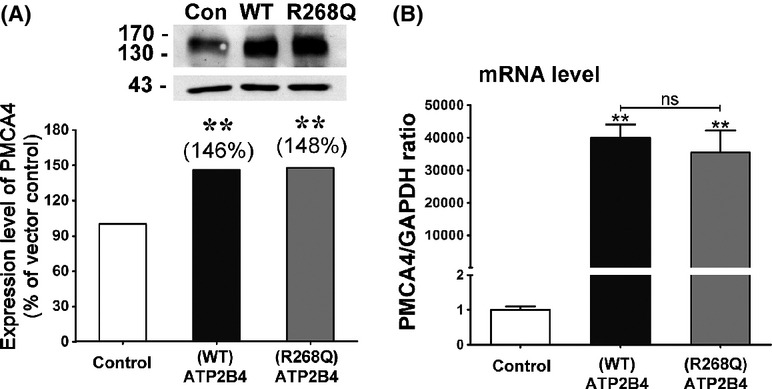
(A) Western blot showing stable overexpression of either wild-type (WT) or R268Q mutant ATP2B4 protein at similar level in SH-SY5Y cells. (B) ATP2B4 mRNA levels in WT and mutant stably overexpressing cells were similar as shown by quantitative real-time PCR. ** represents statistical significance at *p*<0.01 as compared to vector. ns: Not statistically significant.

**Figure 2:**
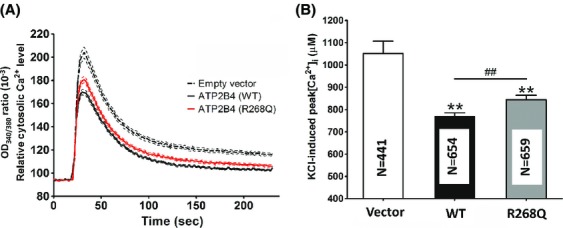
(A) Real-time plot of changes in cytosolic calcium level in SH-SY5Y cells overexpressing either WT or R268Q mutant ATP2B4 by KCl-induced depolarization. Cells were labeled with 5 *μ*mol/L fura2-AM fluorescent dye in serum-free DMEM for 20 min at room temperature. Relative changes in intracellular calcium levels [Ca^2+^]_i_, indicated as normalized OD_340/380_ ratio were plotted against time. Dotted lines represent 95% confident intervals (CI) of the corresponding curves. (B) Maximum cytosolic calcium surge indicated as KCl-induced peak [Ca^2+^]_i_ of individual cells within the monitored period are shown in bar chart. *N* = Number of individual cells measured in each group. Data represent mean ± SD. Comparison between groups was performed using unpaired *t*-test. R268Q mutant ATP2B4 significantly delayed attenuation of KCl-induced calcium surge compared to wild-type. **represents statistical significance as compared with vector controls at *P* < 0.01; ^##^represents *P* < 0.01 between the two designated groups.

**Figure 3:**
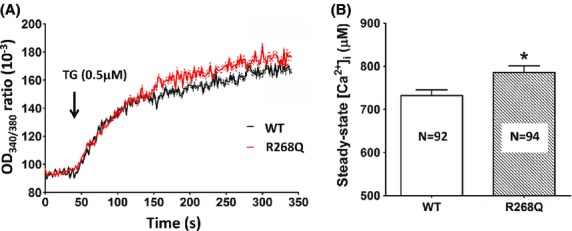
(A) Real-time plot of changes in cytosolic calcium level in SH-SY5Y cells overexpressing either WT or R268Q mutant ATP2B4 by SERCA inhibition using thapsigargin. Cells were labeled with 5 *μ*mo/L fura2-AM fluorescent dye in serum-free DMEM for 20 min at room temperature. Relative changes in intracellular calcium levels [Ca^2+^]_i_, indicated as normalized OD_340/380_ ratio were plotted against time. (B) The mean of maximum steady level of cytosolic calcium of individual cells within the monitored period are shown in bar chart. *N* = Number of individual cells measured in each group. Data represent mean ± SD. Comparison between groups was performed using unpaired t-test. Cells overexpressing R268Q mutant protein demonstrated significantly higher cytosolic calcium concentration compared to wild-type. *represents statistical significance as compared with WT at *P* < 0.05.

To assess the cellular functional impact of the p.R268Q mutation on *PMCA4*, we overexpressed either wild-type or mutant PMCA4 protein in human SH-SY5Y cells and assessed changes in intracellular calcium transient. Overexpression of either wild-type or mutant PMCA4 was confirmed by western blotting using antibodies against PMCA4 and *β*-actin (Fig.1A), and real-time RT-PCR (Fig.1B). To assess the efficiency of intracellular calcium extrusion, addition of KCl was used to induce membrane depolarization and calcium transient. KCl treatment caused immediate increase in [Ca^2+^]_i_ in all test groups, as indicated by a rapid increase in OD_340/380_ ratio under confocal microscopy (Fig.2A). Cells overexpressing either wild-type or mutant PMCA4 had lower maximum [Ca^2+^]_i_ than empty-vector control (*P* < 0.01) (Vector controls: 1051.87 ± 54.9 *μ*mol/L; PMCA4(WT): 769.0 ± 16.1 *μ*mol/L; PMCA4(R268Q): 843.9 ± 20.6 *μ*mol/L) (Fig.2B), suggesting that overexpression of either wild-type or mutant PMCA4 both increased calcium extrusion activity. However, R268Q mutant cells demonstrated significantly higher maximum calcium surge compared to wild-type (all *P* < 0.0001) (Fig.2A). After the initial influx, OD_340/380_ ratio was gradually restored back towards basal level by plasma membrane calcium transporters. There was no statistical difference in time-dependent decay (*t*_1/2_) of calcium surge between cells overexpressing WT (*t*_1/2_ = 23.06) and mutant PMCA4 (*t*_1/2_ = 22.26), (95% confidence intervals: WT = 22.36–23.80; R268Q mutant = 21.51–23.07; *P* = 0.1502) based on extra sum-of-squares *F* test in one phase decay analysis. To further elucidate the contribution of PMCA4 to the calcium transient, we performed additional experiments to measure the steady state [Ca^2+^]i after incubation with SERCA inhibitor, thapsigargin (TG; 500 nmol/L) (Fig.3A). The steady-state [Ca^2+^]i in cells overexpressing mutant PMCA4 after exposure to TG was significantly higher than cells overexpressing WT PMCA4 (WT: 732.3 ± 13.0 *μ*mol/L vs. R268Q: 785.4 ± 16.1 *μ*mol/L; *P* < 0.05; *n* > 92) (Fig.3B).

## Discussion

Calcium is important in controlling vital processes in cells, and its movement and concentration must be tightly regulated. Dysregulation of calcium homeostasis has been shown to be involved in neurodegenerative conditions such as Alzheimer's disease and Parkinson's disease (reviewed in Zundorf and Reiser 2011). In the plasma membrane, there are two calcium exporting systems: the Na^+^/Ca^2+^ exchanger which functions as a large capacity, poor Ca^2+^ affinity system to dispose of periodic arrival of large Ca^2+^ loads, and the PMCAs, which are only present in small amounts, have high Ca^2+^ affinity, and are thought to be important in the fine-tuning of Ca^2+^ signals (Giarcomello et al. 2013).

PMCA4 belongs to the family of plasma membrane Ca^2+^-ATPases consisting of four isoforms with dozens of variants generated by alternative RNA splicing (Strehler and Zacharias 2001). Once thought to have general house-keeping functions in extruding calcium from cells, the PMCAs are now thought to have more specialized roles in the regulation of the extent, localization, and magnitude of calcium movement across the plasma membrane, as accumulating evidence has shown that the expression and localization of different isoforms and splice variants of PMCAs are tightly regulated (Giarcomello et al. 2013). PMCAs have been reported to be associated with the pathogenesis of various human disorders. Pump dysregulation of nongenetic origin has been associated with cancer, and potential therapeutic opportunities by developing agents that modulate PMCA activity are being explored (Brini et al. 2013). Pump defects of genetic origin have also been described in PMCA2 and PMCA3, mutations of which cause hereditary deafness and X-linked cerebellar ataxia in humans, respectively (Strehler 2013). Our previous finding of a PMCA4 mutation causing FSP expanded the spectrum of phenotypes associated with PMCA mutations in humans.

The expression of PMCA4 is highly regulated temporally and spatially. During development, mRNA of PMCA4 is expressed at low levels except in the liver. At later developmental states, its expression in the liver decreases while it increases in the nervous system and a few other tissues. In adult rats, PMCA4 is expressed ubiquitously, most notably in the brain, the heart, and the spermatozoa (Giarcomello et al. 2013). Even within the adult brain, expression of different splice variants of PMCA4 is highly regulated: the full length variant *b* is present in various regions including the cerebellum, whereas the C-terminally truncated variant *a* is most abundant in the frontal cortex (Filoteo et al. 1997). This specialization in the expression of different PMCA4 variants suggests that they likely serve specific functions in different regions of the brain.

Of the 4 PMCA isoforms, PMCA4 is the only one which is localized in lipid rafts in pig cerebellum (Sepulveda et al. 2006). Lipid rafts are specialized lipid domains containing sphingolipids and cholesterol which provide a platform for the assembly of protein complexes involved in signal transduction. They are found in neuronal dendrites where postsynaptic protein complexes are localized. Thus, localization of PMCA4 in lipid rafts suggests that it may play a role in signaling pathways at synaptic nerve terminals, where the synaptic activity is highly dependent on calcium signaling (Simons and Toomre 2000). Lipid rafts are also involved in conformational changes in proteins underlying the formation of amyloid plaques in Alzheimer's disease and prion diseases (Fantini et al. 2002). Mutations in PMCA4 may therefore contribute changes in lipid raft functions leading to neurodegeneration.

In this study, we showed that the R268Q mutation of the PMCA4 gene had functional implication and resulted in increased maximum KCl-induced calcium transient. Even though the absolute difference of the two peak ratios between WT and mutant PMCA4 appeared to be small at 74 *μ*mol/L, the difference was statistically significant with measurements from more than 600 individual cells in three independent cultures. We have also shown that even after controlling for the contribution of SERCA in cytosolic calcium concentration, the delay and impairment in calcium extrusion remained significant in cells overexpressing mutant PMCA4 compared with WT. Considering that the typical basal [Ca^2+^]_i_ is maintained at submicromolar level, this small difference may be sufficient to cause significant differences in the many downstream calcium-sensitive intracellular signaling pathways (Nutt et al. 2002). Moreover, the transient accumulation of free Ca^2+^ (calcium overload) between neuronal excitation in cells overexpressing mutant PMCA4 may result in subsequent activation of various cell death pathways, for example, Ca^2+^-dependent synthases and proteases to damage cytoskeleton, membrane, and DNA leading to excitotoxicity and neuronal death (Gleichmann and Mattson 2011). Indeed, previous work with mutant PMCA2 associated with hereditary deafness in humans has shown that the mutant pumps were defective in the removal of calcium from the cytosol (Giacomello et al. 2011). Mutation in PMCA3, which was found in a family with congenital X-linked cerebellar ataxia, was also found to reduce the ability of the PMCA3 pump to return calcium level to baseline after stimulation of calcium influx (Zanni et al. 2012). Thus, it is likely that the R268Q mutation in PMCA4, which causes functional impairment in calcium extrusion similar to mutated PMCA2 and PMCA3, plays a role in the pathogenesis of the clinical phenotype of FSP.

Taken together, we believe that the R268Q mutation in PMCA4 caused neuronal deficits associated with FSP. This is the first report to demonstrate a PMCA4 mutation which caused functional changes in calcium extrusion to be associated with autosomal dominant FSP, indicating that calcium dysregulation may be involved in the pathogenesis of spastic paraplegia. The detailed pathogenic mechanisms of how impairment in neuronal calcium flux can directly cause the disease phenotype in FSP require further studies.
